# Voluntary Elbow Extension-Flexion Using Single Joint Hybrid Assistive Limb (HAL) for Patients of Spastic Cerebral Palsy: Two Cases Report

**DOI:** 10.3389/fneur.2019.00002

**Published:** 2019-01-22

**Authors:** Yukiyo Shimizu, Hideki Kadone, Shigeki Kubota, Tomoyuki Ueno, Yoshiyuki Sankai, Yasushi Hada, Masashi Yamazaki

**Affiliations:** ^1^Department of Rehabilitation Medicine, University of Tsukuba Hospital, Tsukuba, Japan; ^2^Center for Innovative Medicine and Engineering, University of Tsukuba Hospital, Tsukuba, Japan; ^3^Division of Regenerative Medicine for Musculoskeletal System, Faculty of Medicine, University of Tsukuba, Tsukuba, Japan; ^4^Center for Cybernics Research, University of Tsukuba, Tsukuba, Japan; ^5^Department of Orthopaedic Surgery, Faculty of Medicine, University of Tsukuba, Tsukuba, Japan

**Keywords:** cerebral palsy, spastic diplegia, hybrid assistive limb (HAL), coactivation index, synergy analysis

## Abstract

Cerebral palsy (CP) patients with spastic diplegia struggle to perform activities of daily life (ADL) using their upper arms. The single-joint-type Hybrid Assistive limb (HAL) for upper limbs is a new portable robot that can provide elbow motion support in accordance with bioelectric activation of patient's biceps and triceps brachii muscles. The purpose of this study is to assess the feasibility and efficacy of the use of HAL for CP patients. Two patients were enrolled in this study. (Case 1: a 19-years-old male, at the Gross Motor Function Classification System (GMFCS) level IV, Case 2: a 17-years-old male at GMFCS level III). Both these patients experienced difficulty in voluntary elbow extension in ADLs. The HAL intervention (eight sessions; voluntary extension-flexion training of the elbow with HAL and clinical evaluation) was conducted for both sides in Case 1 and for the right side in Case 2. Clinical assessments were conducted as follows: Surface electromyography was used to evaluate the muscle activities of the biceps, triceps brachii, trapezius, and pectoralis major during elbow extension-flexion. The voluntary extension-flexion angles of the elbow, the coactivation index of the biceps and triceps brachii muscles, synergy analysis, and the Action Research Arm Test (ARAT) scores were assessed before and after the HAL sessions; the FIM score was evaluated before and after the entire intervention. In Case 1, the voluntary extension angle tended to increase after the HAL sessions. In both cases, the ARAT scores improved after the sessions. The FIM scores improved after HAL intervention. The voluntary extension-flexion of the elbow using the HAL may be a feasible option for rehabilitation of CP patients.

## Introduction

Cerebral palsy (CP) is a major cause of childhood disability (1). Although perinatal medical care is progressing, CP has remained prevalent in recent years, along with the increased survival of at-risk preterm infants (1–4).

Spastic diplegia represents the most common type of CP seen in premature infants. It features a disproportionate disorder of the lower limbs, although upper limb abnormalities, manifested as motor perceptual dysfunction, are very common as well (5). Patients of spastic diplegia struggle to perform the activities of daily life (ADL) using their upper arms due to weakness and the abnormal tonus of the relevant muscles. These upper limb impairments lead to difficulties in reaching, grasping, and manipulating objects. Improving upper limb motion may enhance the patients' ability to perform ADLs and, thus, the quality of their lives.

Past studies in this domain have reported on the excessive coactivation in patients of CP (6–8). Coactivation, which is the concurrent activation of agonist and antagonist muscles around a joint (9) is one reason for the weakness of muscles around a joint (6) or motor control disorder in patients of spastic CP patents (7). Coactivation has also been reported to cause a reduction in the active range of motion of the elbow (8).

Synergy analysis is used to assess the efficacy of rehabilitation therapies (10–13). It can be used as a physiological marker in patients suffering from stroke or trauma (10), and in patients of CP as a tool to monitor motor impairments, such as those related to coordination (11). It can evaluate a slight recovery in stroke patients (12) and has been used as an evaluation tool for robotic intervention (13).

The single-joint-type hybrid assistive limb (HAL^®^) (HAL^®^-SJ; Cyberdyne Inc., Ibaraki, Japan) was developed to support the motion of joints of the elbow or knee. A small power unit on the lateral side of the joint consists of angular sensors and actuators, and the primary control system consists of cybernic voluntary control (CVC) based on the intention of the motion by using bioelectric signals generated by the patient's muscle activities (14). We previously reported on upper limb HAL-SJ for voluntary elbow flexion in a patient with complete quadriplegia due to a spinal cord injury (14), a patient with postoperative brachial plexus palsy (15), and a patient with postoperative C5 palsy (16).

We had hypothesized that the use of the HAL for upper limb may reduce the coactivation of elbow flexors and extensor or change muscle synergy of the upper limbs.

The aim of this study is to describe the feasibility and efficacy of upper limb training using HAL-SJ for CP patients with spastic diplegia. To the best of the authors' knowledge, this is the first report on the use of HAL-SJ for patients with CP.

## Participants and Methods

### Participants

Two patients were enrolled for this study. Case 1 corresponded to a 19-years-old male, who had been born prematurely at a very low birth weight (1,350 g at a 28 weeks 4 days' gestational age), classified as Gross Motor Function Classification System (GMFCS) (17) level IV and the Manual Ability Classification System (MACS) (18) level III. He also had visuoperceptual function disorder owing to oculomotor disturbance. Case 2 corresponded to a 17-years-old male, also prematurely born with a low birth weight (1,861 g at 31 weeks 0 days' gestational age), classified as GMFCS level III and MACS level II. Both had difficulty in voluntary elbow extension in ADLs, such as changing clothes.

### HAL Intervention (Figure 1, Supplementary Video 1)

The HAL device consisted of two links and one electric actuation unit embedded on the joint connecting the links. Each of the links was equipped with a supporter and belts to be tightened on the patient's upper limb; one on the upper arm and the other on the forearm, so that the links and the joint are aligned with the upper arm, forearm and elbow joint on the lateral side. The surface electrodes of HAL for elbow flexion was attached to the biceps and those for extension was attached to triceps brachii. HAL's assistance torque was computed by weighted difference of the activation of these muscles. In equation, T = Gf ^*^Af – Ge ^*^Ae, where T is the assistance torque, Gf and Ge are gain parameters, and Af and Ae are filtered activation of the flexion and extension muscles. The gain parameters were adjusted manually in each session to achieve a wide range of flexion and extension motion while maintaining comfort.

**Figure 1:**
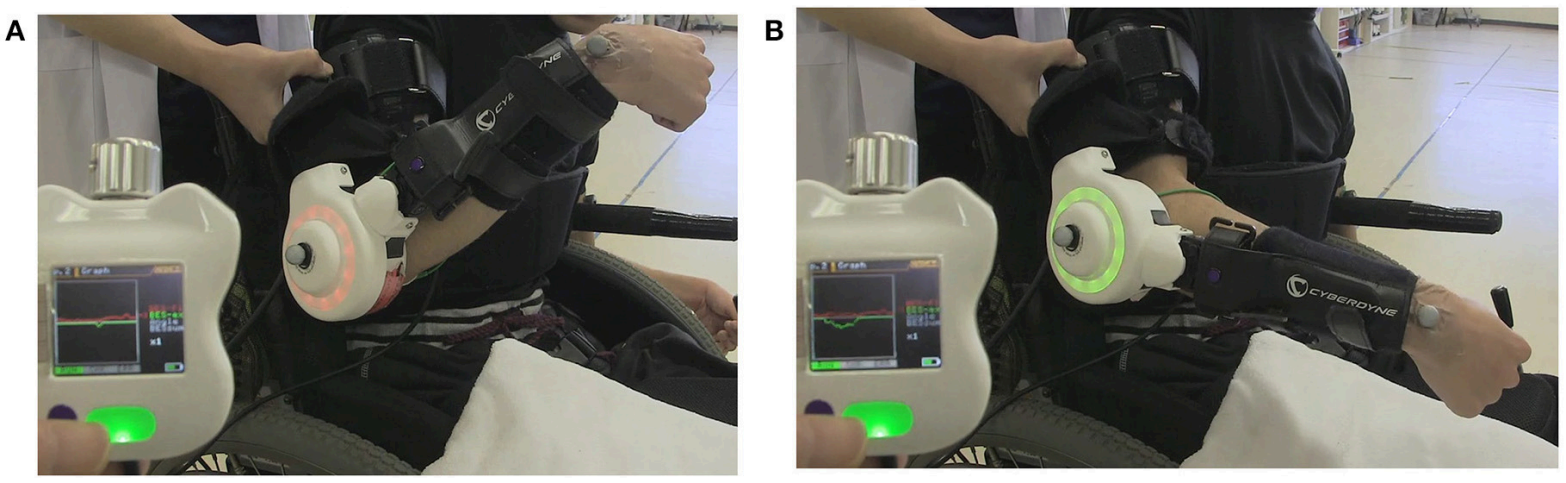
The first HAL intervention in Case 1. During the session, a therapist supported the patient's arm. The display of the controller shows bioelectric signals from the right bicep for elbow flexion as a red line, and those from the triceps for elbow flexion as a green line. The HAL elbow joint flashed a red light when the HAL was flexed and green when extended. **(A)** Voluntary flexion was intended with the HAL. **(B)** Extension was intended.

The HAL intervention constituted eight sessions. Each consisted of voluntary extension-flexion training of the elbow using the HAL and clinical evaluation. The intervention was conducted for the both sides in Case 1 and for the right side in Case 2.

The frequency of sessions ranged from 1 to 3 per month. In both cases, HAL intervention was performed on an outpatient basis in addition to standard physical or occupational therapy, which was implemented 1–2 times per month.

Each session with the HAL lasted 60 min, including rest and the time required to attach and detach the device (10 min to attach and five to detach). The remaining time was allocated as follows: approximately 30 min for elbow flexion and extension exercises, including rest, and 15 min for evaluation before and after HAL intervention. A physiatrist was on staff and was present in case of an emergency. A therapist and a co-operator attached and detached the HAL, and an engineer implemented motion analysis.

### Clinical Assessments

Clinical assessments were conducted before, during, and after the intervention. The maximum extension and flexion angles during voluntary elbow movements were calculated as the static range of motion (ROM). We also calculated the sum of the modified Ashworth scale (MAS) scores (19) in each upper limb (range: 0–28; shoulder flexion, abduction, elbow flexion, extension, forearm supination, and wrist flexion and extension) before and after each session.

We performed the elbow extension-flexion test five times before and after HAL to assess the motion of the elbow joint. The TrignoTM Lab Wireless electromyography (EMG) system (Delsys Inc., Boston, MA, USA) was used to evaluate the muscular activity of the biceps and triceps brachii, and the trapezius, and pectoralis major during elbow flexion and extension. Motion capture (VICON MX with 16 T20S cameras; VICON, UK) was used to record upper limb motion and obtain the dynamic elbow ROM in synchronization with electromyography. Autoreflective markers were placed bilaterally on the acromion, the humeral lateral epicondyle, and the distal radioulnar joint. The elbow extension and flexion phases were extracted according to the trajectory of the markers. The activity of each muscle was evaluated by EMG collected at 2,000 Hz and filtered using passing filter with a bandwidth of 30–400 Hz. An activation envelope was computed by a 200-ms moving window average using scripts on MATLAB 8.2 (MathWorks, Natick, MA, USA). We also calculated the coactivation index (CAI) of the voluntary elbow angle during active elbow extension and flexion, which were computed by the un-centered Pearson correlation of the envelope profiles of the biceps and triceps muscles. Moreover, we used muscle synergy analysis with non-negative matrix factorization (NNMF) to evaluate the patients' EMG data (10–13), and compared the variance accounted for (VAF) against each of the possible number of synergy modules (1, 2, 3, and 4) bimanually before and after each session.

Scores for voluntary elbow extension, flexion angles, and MAS scores, CAI, and VAF, before and after each HAL session were compared using the Wilcoxon signed-rank test. All statistical analyses were performed using the JMP^®^ 13.0.0 (SAS Institute Inc., Cary, NC, USA); *P* < 0.05 were considered significant.

For post-experiment evaluation of HAL intervention, the Action Research Arm Test (ARAT) (20–22) was performed before and after the HAL in both cases. The ARAT is among the most widely used standardized measures for the upper limbs; it is efficient and can assess both the arm and the hand during the execution of functional tasks. It contains assessment of four groups of motion (grasp, grip, pinch, and gross movements) that incorporate some bimanual ADLs. Functional independence measurement (23, 24) was also used to assess the change in patients' performance of ADLs.

## Results

Both participants completed all sessions. The only observed adverse effect of the experiments was redness caused by contact between the skin and the forearm supporter, which soon disappeared.

Table 1 shows changes in the static and dynamic, voluntary, maximum elbow extension, and flexion angles. The increase in the extension angles was statistically significant from before to after the right HAL session of Case 1 (from −38.1 ± 8.4 to −25.0 ± 1.9; *P* = 0.031, from −78.1 ± 15.2 to −62.2 ± 9.1; *P* = 0.078, respectively). The dynamic voluntary elbow extension angles tended to increase from before to after the right HAL session of Case 1, and the decrease in the voluntary elbow flexion angle was statistically significant from before to after the left HAL session in Case 1 (from 141.1 ± 8.1 to 135.9 ± 8.3; *P* = 0.016). Statistical significance was not observed in the other angle evaluations (Supplementary Tables 1,2).

**Table 1:** Change in static and dynamic maximum voluntary elbow extension and flexion angle in Right-sided HAL in Case 1.

	**Static ROM**		**Dynamic ROM**	
	**Rt**	**Lt**	**Rt**	**Lt**
Pre-extension	−38.1 ± 8.4	−31.9 ± 10.0	−78.1 ± 15.2	−81.2 ± 13.5
Post-extension	−25.0 ± 1.9	−24.3 ± 8.4	−62.2 ± 9.1	−73.9 ± 19.9
	^*^P = 0.031	NS	NS (*P* = 0.078)	NS
Pre-flexion	141.9 ± 6.5	136.9 ± 11.9	143.3 ± 6.2	141.1 ± 8.1
Post-flexion	145.0 ± 2.9	140.7 ± 5.3	144.9 ± 4.5	135.9 ± 8.3
	NS	NS	NS	^*^P = 0.016

*Asterisk means that the probability level was accepted for statistical significance, P < 0.05*.

**Figure 2:**
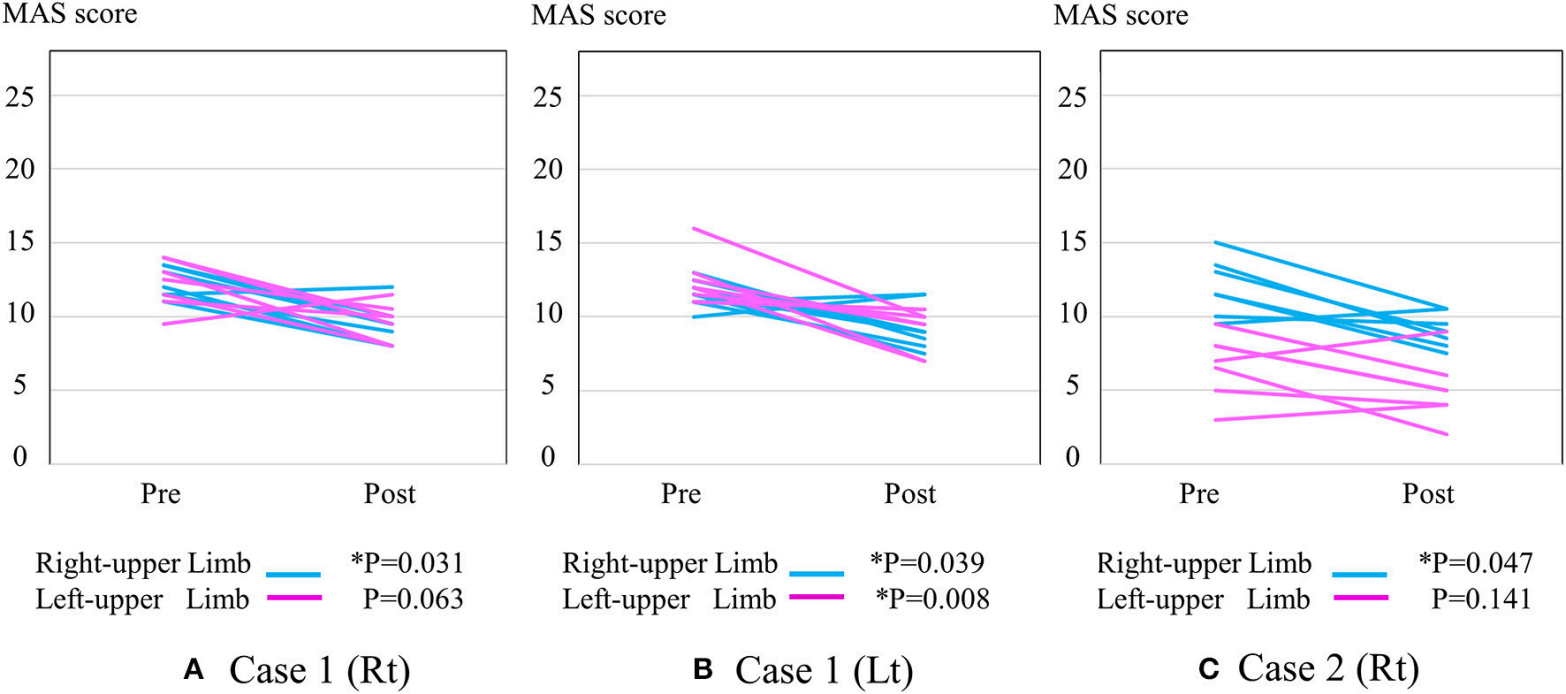
The change in total MAS score. **(A)** Right-sided HAL in Case 1 **(B)** Left-sided HAL in Case 1 **(C)** Right-sided HAL in Case 2. Y-axis shows total MAS score of upper limb joints.

Figure 2 shows the change in MAS score. Each post-session MAS score decreased compared with the pre-session MAS score of the right arm in the right HAL session of Case 1 (from 12.3 ± 1.0 to 9.4 ± 1.4; ^*^P = 0.031), the left arm in the right HAL session of Case 1 (from 12.2 ± 1.7 to 9.6 ± 1.3; *P* = 0.063) (Figure 2A), the right arm in the left session of Case 1 (from 11.5 ± 0.9 to 9.3 ± 1.5; ^*^P = 0.039), the left arm in the left session of Case 1 (from 12.4 ± 1.6 to 9.3 ± 1.4; ^*^P = 0.008) (Figure 2B), the right arm in the right HAL session of Case 2 (from 12.0 ± 2.0 to 9.1 ± 1.2; ^*^P = 0.047), and the left arm in the right HAL session of Case 2 (from 6.7 ± 2.2 to 5.0 ± 2.2; *P* = 0.141) (Figure 2C).

**Figure 3:**
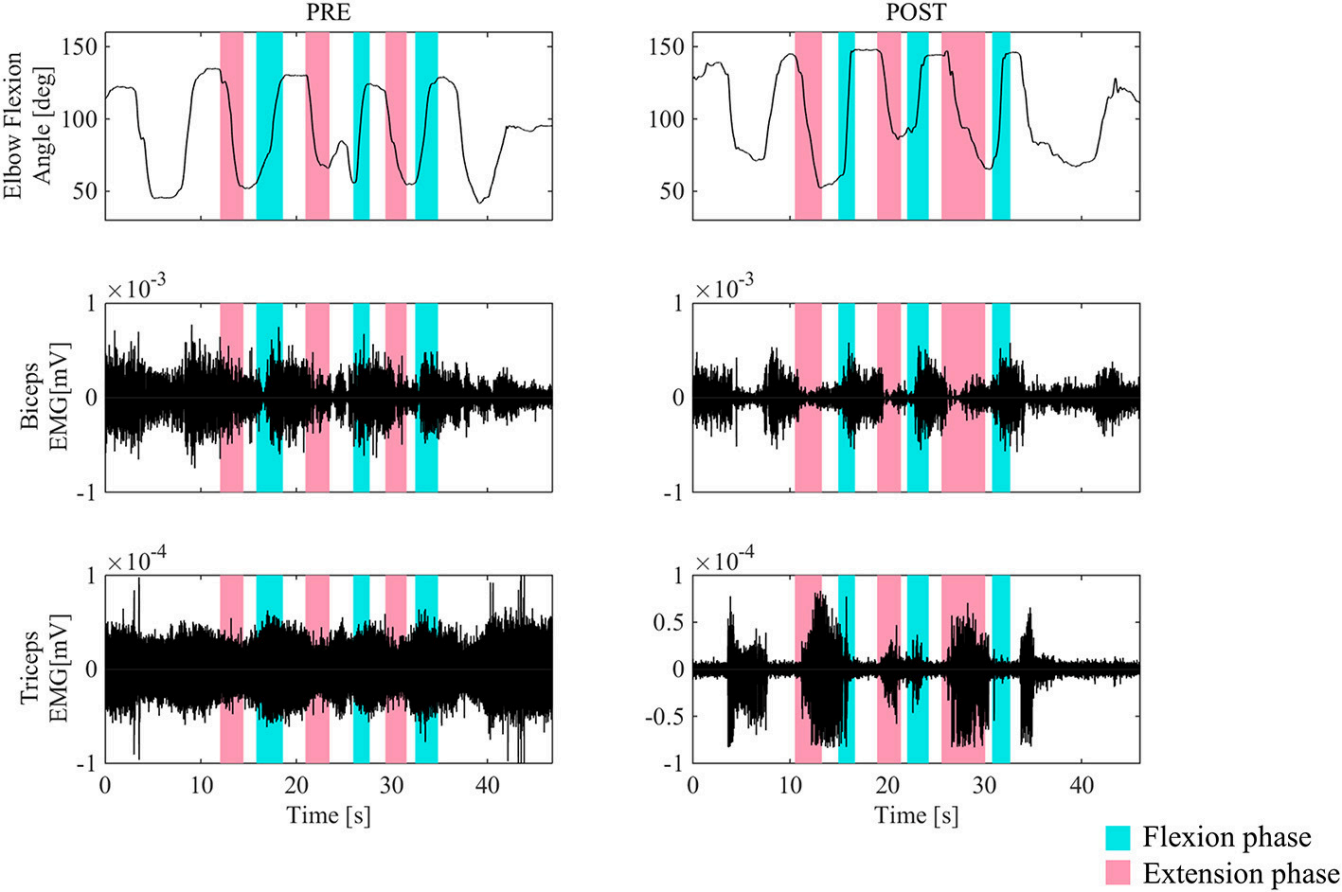
The surface EMG of the biceps and triceps brachii during elbow flexion (blue) and extension (red) for the right arm before and after the second HAL session for the right elbow in Case 1. Before the session, a coactivation of the biceps and triceps was observed in the elbow extension phase. After the session, the triceps muscle was activated in the extension phase dominantly. Periodic activation of these muscles was noted.

Figure 3 shows the surface EMG of the biceps and triceps brachii for the right arms during elbow flexion and extension without HAL, before and after the second HAL session for the right elbow in Case 1. Before the session, a coactivation of the biceps and triceps was observed in the elbow flexion phase. The biceps stayed activated both in the flexion and extension phases, and instead, the triceps muscles were activated in the flexion phases. However, following the session, the basic contraction of the triceps muscle was reduced, and both muscles exhibited regulated and periodic behavior; the biceps activated dominantly during flexion while the triceps activated during extension.

**Figure 4:**
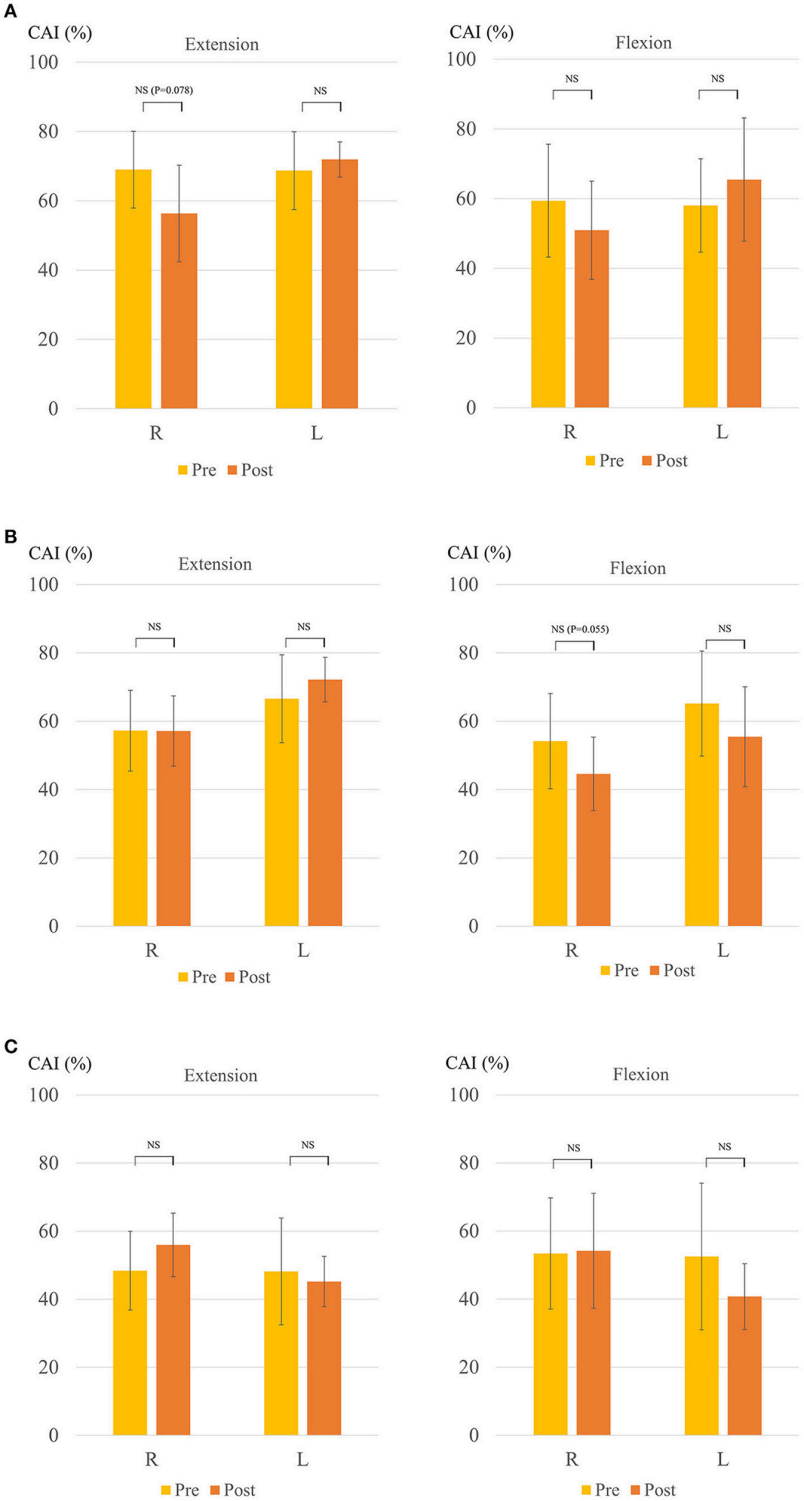
Change in coactivation index (CAI) **(A)** Right-sided HAL in Case 1. **(B)** Left-sided HAL in Case 1. **(C)** Right-sided HAL in Case 2. Y-axis shows CAI (%). The right side shows the extension and the left shows the flexion in each figure.

Figure 4 shows the change in the CAI. The mean CAI of the right biceps and triceps brachii muscles during elbow extension tended to decrease after the HAL for the right side in Case 1 (from 69.0 ± 11.9 to 56.4 ± 14.9; *P* = 0.078) (Figure 4A), as did that of the right during elbow flexion for the left side in Case 1 (from 54.2 ± 14.9 to 44.6 ± 11.5; *P* = 0.055) (Figure 4B). The CAI score in Case 2 was approximately 50 even before the HAL. Thus, there was no statistically significant difference before and after the HAL (Figure 4C).

**Figure 5:**
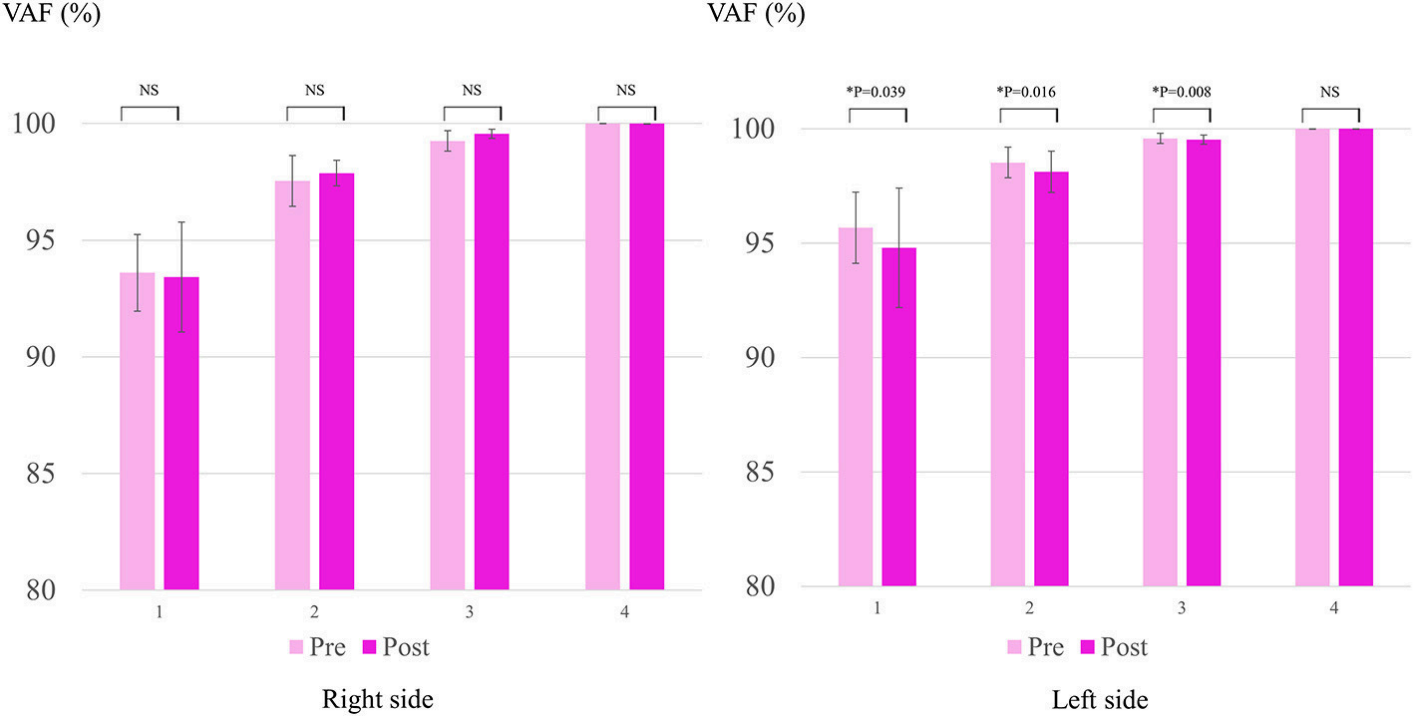
Change in VAF (variance accounted for) in Case 2. Y-axis shows VAF (%).

Figure 5 shows the change in VAF in Case 2. The mean VAF of the left-upper limb decreased after the HAL for the right side, for modules 1, 2, and 3, but not for module 4. There was no statistically significant difference in terms of VAF in the right sides in Cases 1 and 2.

The results of the ARAT are shown in Table 2. In both cases, the scores improved for both sides, including the pinch movement, which involves skilled motor behavior. In both cases, slight changes to the FIM scores (Table 3) were noted, particularly in the dressing scores. Improvements were observed in Case 1 from 2 to 3 in the dressing of lower body, and in Case 2, from 5 to 6 in the upper body and from 4 to 5 in the lower body. Improved independence and reduced need for care in the dressing activity was observed in daily life of the cases.

**Table 2:** The results of ARAT before and after the HAL sessions.

		**Case 1 (Lt HAL)**		**Case 2 (Rt HAL)**	
		**Rt**	**Lt**	**Rt**	**Lt**
Grasp	Pre	9	7	5	17
	Post	14	8	6	18
Grip	Pre	5	3	0	9
	Post	6	3	7	12
Pinch	Pre	1	0	0	6
	Post	1	0	0	15
Gross movement	Pre	9	9	4	9
	Post	9	9	9	9
Total	Pre	24/57	19/57	9/57	41/57
	Post	30/57	20/57	22/57	54/57

**Table 3:** Change in FIM scores.

	**Case 1**		**Case 2**	
	**Pre**	**Post**	**Pre**	**Post**
Eating	5	5	6	6
Grooming	2	2	6	6
Bathing/Showering	2	2	3	3
Dressing upper body	2	3	3	4
Dressing lower body	1	1	3	4
Toileting	1	1	5	6
Bladder management	7	7	7	7
Bowel management	7	7	7	7
Bed/Chair/Wheelchair transfers	2	2	6	6
Toilet transfers	2	2	6	6
Shower/Bath transfers	2	2	4	4
Wheelchair	6	6	6	6
Stairs	1	1	4	4
Comprehension	7	7	7	7
Expression	7	7	7	7
Social interaction	7	7	7	7
Problem solving	7	7	7	7
Memory	7	7	7	7
Total	75	76	101	104

## Discussion

In this study, the HAL-SJ was used to improve voluntary elbow extension–flexion for patients of CP with spastic diplegia. The HAL-SJ is a portable device, which rendered it convenient for use in a clinical setting.

Many patients with CP suffer from spasticity. With regard to spasticity, in both cases, there was an immediate decrease in the MAS in the arm subjected to the HAL after each session. In Case 1, the MAS score decreased after the sessions. Reduction of MAS after HAL was commonly observed in our previous reports on gait training using HAL for patients with spinal cord injury (25, 26). Spasticity negatively influences quality of life (QOL) for many CP patients by the restriction of ROM (27), movement disorder (28) and restricted ADLs. In this sense, elbow extension-flexion training using HAL may be useful to reduce spasticity and to improve their ADLs and QOL.

CAI evaluation shows that the coactivation of the biceps and triceps tended to decrease in the right arm following the application of the HAL to the right side and then to the left side, and the biceps and triceps muscles on both sides began to be activated separately during and after the HAL. Along with this, the angles of static and dynamic extension increased after the HAL. The improvement in the active extension angle might have been accompanied by a decrease in the CAI, as in a previous study that reported a reduction in the activation of the antagonist muscle of the active ROM of the elbow in children with spastic hemiplegic CP (8).

A previous study reported that synergy analysis can help investigate the recovery of upper limb function of subacute stroke patients after robot assisted training (12). In the patients in this study with spastic diplegia CP, VAF on the contralateral side demonstrated interesting changes in Case 2, whereas the CAI recorded no statistically significant difference. Following the HAL, the VAF reduced in the smaller number modules. This indicated that the four measured muscles (biceps, triceps brachii, trapezius, and pectralis major) gained separated control following the HAL and their synergies became applicable to more complex movements requiring a greater number of modules. This is synchronous with Case 2's demonstration of separated movements following the HAL in contrast to that before the HAL, as represented by the improvement in the ARAT scores for both sides, predominantly in the contralateral side. Moreover, the FIM scores improved, which reflected an improvement in the patients' ability to dress themselves in both cases.

Regarding the performance of the upper limbs in diplegic children, Turconi et al. reported that the use of Armeo^®^ Spring is effective for improving coordination, fluency, and dexterity (29). They also claimed that motivation is critical for a patient's compliance to training, and this technology can be effective for intervention. On the other hand, the HAL is a wearable robotic device that can assist with movements according to the wearer's volition (30). Even if there is coactivation in the biceps and triceps brachii, the HAL can be adjusted to regulate the extent of assistance to cut hyper-signals of the activity of the antagonistic muscles. Therefore, wearers can move their joints smoothly at will.

We hypothesize HAL's effect for assisting motor learning process to achieve a smooth volitional motion. Previous studies that have described the critical principles of motor learning for the plasticity of the central nervous system state five characteristics: near-normal movements, movement driven by muscle activation, focused movement, repetition of desired movements, and training specificity (14, 31). HAL's implementation of joint motion assistance, where assistive joint torque is applied in real-time in accordance with activation of the relevant muscles, together with appropriate adjustment of the weight parameters of the agonist and antagonist muscles, helps to achieve these characteristics during remarkably simple but intuitive motion of elbow flexion and extension. Therefore, it may be able to assist motor learning.

Some improvements were seen after the sessions in both sides of the patients, as shown by the kinematic and kinetic analyses and the ARAT assessment, which demands bimanual movements, even if one side was not involved in the HAL. A past study demonstrated that simple unimanual training is effective when learning bimanual motor skills and discussed structured motor learning transfer between unimanual and bimanual movements (32). The motion using HAL, derived from the volitional contraction of the muscles—even if it is extremely low—may influence the central nervous system. Although improvements in the voluntary elbow extension angle and ARAT scores were observed in both cases in this study, the mechanism underlying these changes are left for future investigation.

While we dealt in this study with spastic diplegia, which is the most common type of CP among full-term infants (1, 33), the HAL-SJ may also be effective for patients of spastic hemiplegia. Children suffering from spastic hemiplegia generally experience various motor and sensory impairments, such as muscle weakness, spasticity, abnormal movements, and sensory dysfunctions, and approximately 50% show greater disability in the upper extremities than the lower ones. Dysfunctions in the upper extremity result in these patients being dependent on others to perform daily activities and leads to a lack of successful social integration (34, 35). The effect of HAL-SJ seen in this study may also apply to hemiplegia CP and may be useful to improve their ADL.

Limitations to this study are that there were only a few cases, and that the follow-up periods were short. Prior to and through the HAL intervention period, the patients had been receiving additional standard physical and occupational therapies. However, their ADLs had plateaued for a few years until the HAL interventions. Therefore, we think that standard therapies had little influence on the cases studied here. In general, it is difficult for adolescents with spastic diplegic CP to improve their ADLs. We think that the improvements of their ADLs obtained after the intervention, even slight, might be meaningful and have some effects for their actual daily living.

## Conclusion

In this study, we investigated the feasibility of rehabilitation of elbow flexion and extension using the single-joint-type HAL on two patients of spastic diplegia with CP and confirmed that it can be implemented safely and yield positive outcomes.

## Ethics Statement

This study was conducted in accordance with the Declaration of Helsinki, with approval from the Ethics Committee of the Tsukuba University Faculty of Medicine (approval no.: H26-22). All participants provided written informed consent for participation and publication, including the use of accompanying images.

## Author Contributions

All authors participated in the design, execution, and analysis of these studies, and have seen and approved the final version of the manuscript. YuS and HK participated in the study design and drafted the manuscript. YuS, HK, and SK executed the HAL interventions and performed the data analysis. YoS conceived of the device and helped draft the manuscript. TU, YH, and MY participated in the study design and helped draft the manuscript. MY was the principal investigator of this study, and participated in its design and coordination.

### Conflict of Interest Statement

YoS is the C.E.O., shareholder, and director of CYBERDINE Inc., which produces the robot suit HAL. CYBERDINE was not involved in the study design, data collection, analysis, writing, or submission of this article. The remaining authors declare that the research was conducted in the absence of any commercial or financial relationships that could be construed as a potential conflict of interest.

## Supplementary Material

Supplementary Table 1 Change in static voluntary elbow extension and flexion angle in Left-sided HAL in Case 1 and Right-ided HAL in Case 2.

Supplementary Table 2 Change in dynamic voluntary elbow extension and flexion angle in Left-sided HAL in Case 1 and Right-sided HAL in Case 2.

Supplementary Video 1 This movie shows the voluntary elbow extension-flexion for the right arm before, during, and after the first HAL session for the right elbow in Case 1.
