# Supplementary material for: Voluntary Elbow Extension-Flexion Using Single Joint Hybrid Assistive Limb (HAL) for Patients of Spastic Cerebral Palsy: Two Cases Report
Source: Front Neurol. 2019 Jan 22;10:2. doi: 10.3389/fneur.2019.00002 (PMC6349701; doi:10.3389/fneur.2019.00002)
Supplement: Supplementary Table 1 — Change in static voluntary elbow extension and flexion angle in Left-sided HAL in Case 1 and Right-ided HAL in Case 2. [file Data_Sheet_1.pdf]

Supplementary Material

1 Supplementary Tables

Supplementary Table 1.

Change in static voluntary elbow extension and flexion angle in Left-sided HAL in Case 1 and Right-sided HAL in Case 2.

	Case 1 (Lt)		Case 2 (Rt)	
	Rt	Lt	Rt	Lt
Pre-extension	-30.0±7.6	-30.6±8.6	-49.4±11.2	-9.4±4.2
Post-extension	-28.8±9.9	-28.8±9.2	-40.7±4.5	-7.1±2.7
	NS	NS	NS	NS
Pre-flexion	142.5±4.6	142.5±2.7	141.9±3.7	136.9±3.7
Post-flexion	145.6±4.2	141.9±5.3	137.9±3.9	139.3±1.9
	NS	NS	NS	NS

Supplementary Table 2.

Change in dynamic voluntary elbow extension and flexion angle in Left-sided HAL in Case 1 and Right-sided HAL in Case 2.

	Case 1 (Lt)		Case 2 (Rt)	
	Rt	Lt	Rt	Lt
Pre-extension	-79.5±11.5	-85.7±12.4	-68.8±9.1	-36.5±4.9
Post-extension	-71.5±21.1	-84.0±17.7	-70.5±8.6	-37.2±5.4
	NS	NS	NS	NS
Pre-flexion	142.2±3.8	134.4±8.9	152.4±5.5	147.2±3.1
Post-flexion	140.4±7.4	135.9±8.7	147.4±7.1	148.8±5.3
	NS	NS	NS	NS
